# Clinical significance of soluble CD163 in polymyositis-related or dermatomyositis-related interstitial lung disease

**DOI:** 10.1186/s13075-016-1214-8

**Published:** 2017-01-19

**Authors:** Yasunori Enomoto, Yuzo Suzuki, Hironao Hozumi, Kazutaka Mori, Masato Kono, Masato Karayama, Kazuki Furuhashi, Tomoyuki Fujisawa, Noriyuki Enomoto, Yutaro Nakamura, Naoki Inui, Daisuke Suzuki, Noriyoshi Ogawa, Ran Nakashima, Tsuneyo Mimori, Toshihide Iwashita, Takafumi Suda

**Affiliations:** 1 Second Division, Department of Internal Medicine, Hamamatsu University School of Medicine, 1-20-1 Handayama, Hamamatsu, Shizuoka 431-3192 Japan; 2 Department of Regenerative and Infectious Pathology, Hamamatsu University School of Medicine, Hamamatsu, Japan; 3 Department of Clinical Pharmacology and Therapeutics, Hamamatsu University School of Medicine, Hamamatsu, Japan; 4 Third Division, Department of Internal Medicine, Hamamatsu University School of Medicine, Hamamatsu, Japan; 5 Department of Rheumatology and Clinical Immunology, Kyoto University Graduate School of Medicine, Kyoto, Japan

**Keywords:** CD163, Myositis, Interstitial lung diseases, Macrophage, Biomarker

## Abstract

**Background:**

Macrophage activation is involved in the pathogenesis of polymyositis (PM)/dermatomyositis (DM). CD163, a scavenger receptor expressed on the surface of activated macrophages, mediates anti-inflammatory functions. This study aimed to evaluate the clinical significance of soluble CD163 (sCD163) in PM/DM-related interstitial lung disease (ILD).

**Methods:**

The main subjects were 48 patients with PM/DM-related ILD. As controls, 10 patients with PM/DM without ILD and 20 healthy volunteers were enrolled. In patients with PM/DM-related ILD, the baseline characteristics and clinical course were obtained through a review of patient medical records. Serum sCD163 levels at ILD diagnosis were quantified by enzyme-linked immunosorbent assay, which were compared with the other baseline clinical factors and evaluated for potential as a prognostic biomarker. In addition, immunohistochemistry analysis using anti-human CD163 antibody was performed on the lung sections of two patients with DM-related ILD (a survivor and non-survivor, respectively) and one patient with early-stage lung cancer as a normal control.

**Results:**

The median value of serum sCD163 in patients with PM/DM-related ILD was 818 ng/mL, which was higher than that of PM/DM patients without ILD and healthy volunteers (716 ng/mL and 340 ng/mL, respectively). Significant but mild correlations with serum sCD163 levels were observed for serum C-reactive protein levels (*r* = 0.322) and % predicted forced vital capacity (*r* = −0.301) in patients with PM/DM-related ILD. A Cox proportional hazard model demonstrated that patients with PM/DM-related ILD and higher sCD163 levels had worse prognosis (age-adjusted and gender-adjusted hazard ratio per 100 ng/mL increase 1.27, 95% confidence interval 1.11–1.45, *P* <0.001). In immunohistochemistry analysis, compared with normal lung, alveolar infiltration of CD163-positive macrophages was evident in the lungs of patients with DM-related ILD. Especially, the finding was more severe in the non-survivor’s lung.

**Conclusions:**

Serum sCD163 might be a potential biomarker for predicting the severity and prognosis of PM/DM-related ILD. Our results suggest the importance of macrophage activation in the disease.

**Electronic supplementary material:**

The online version of this article (doi:10.1186/s13075-016-1214-8) contains supplementary material, which is available to authorized users.

## Background

Polymyositis (PM) and dermatomyositis (DM) are systemic inflammatory diseases affecting the muscles, skin, and several other organs including the lungs [1, 2]. Interstitial lung disease (ILD) is a common complication seen in 20–78% of patients with PM/DM and can be a prognostic determinant [3–8]. Particularly, patients with DM and clinically amyopathic DM (CADM) frequently develop an acute and severe form of ILD [4, 5]. Thus, management of ILD is essential for the care of patients with PM/DM, and appropriate biomarkers reflecting the disease severity are required.

CD163, a scavenger receptor specifically expressed on the surface of activated macrophages and monocytes, is involved in the uptake of hemoglobin-haptoglobin and mediates the clearance of hemoglobin [9]. CD163 expression is upregulated by several inflammatory cytokines including interleukin (IL)-6 [10, 11]. Inflammatory infiltrates containing CD163-positive macrophages are frequently found in the diseased muscles in PM/DM [12, 13], where activated macrophages themselves directly induce inflammation and also mediate inflammatory responses via lymphocyte activation [14, 15]. Indeed, PM/DM is one of the diseases that occasionally cause macrophage activation syndrome (MAS). In contrast, macrophages could produce transforming growth factor-β, the most prominent fibrosis-inducing molecule, in chronic lung inflammation [16]. Additionally, activated macrophages expressing CD163 have been demonstrated to play a key role during lung fibrosis [17]. Macrophage activation would be involved in the pathogenesis of PM/DM-related ILD.

Upon acute and chronic inflammations, CD163 is shed from surface of activated macrophages and released into peripheral blood in the soluble form [11]. Therefore, soluble CD163 (sCD163) in the serum has been highlighted as a representative of macrophage activation and as a biomarker in several inflammatory diseases [18–23]. However, the clinical significance of this molecule in PM/DM-related ILD is not fully understood. The aim of this retrospective study was to evaluate the potential of serum sCD163 as a biomarker in PM/DM-related ILD.

## Methods

### Patient selection

A retrospective review of 52 patients with PM/DM-related ILD, consecutively admitted to Hamamatsu University Hospital between 1995 and 2014, was conducted. Subsequently, four patients (one with PM, one with CADM, and two with classic DM) who received immunosuppressive treatment with corticosteroids on diagnosis of ILD were excluded. Thus, this study included the remaining 48 patients with PM/DM-related ILD. Among them, 45 patients had never received immunosuppressive treatment, whereas the other 3 patients had received treatment but terminated it at least 1 year before the ILD diagnosis. In addition, 10 patients with PM/DM without ILD, who had not received immunosuppressive treatment, and 20 age-matched and gender-matched healthy volunteers were enrolled as control groups (Additional file 1: Table S1).

Classic PM/DM was diagnosed according to the criteria of Bohan and Peter [2]. Patients with definite or probable PM/DM according to these criteria were included in the study. CADM was diagnosed when a patient had a skin rash typical of DM without clinical evidence of myositis and with little or no increase in serum creatine kinase (CK) [5, 24]. The diagnosis of classic PM/DM and CADM had been confirmed by the dermatologists and/or rheumatologists and the pulmonologists in our institution. The diagnosis of ILD was based on the radiological assessment of high-resolution computed tomography (HRCT) of the chest, which included appearances of bilateral reticulation, ground-glass attenuation, or consolidation.

### Data collection

The patients’ clinical data at the time of PM/DM-related ILD diagnosis were obtained through medical record reviews. These data included symptoms; laboratory findings; pulmonary function test results (forced vital capacity (FVC); diffusing capacity of the lung for carbon monoxide (DLco), measured using a single breath technique); analyses of bronchoalveolar lavage (BAL) fluid; treatment; and prognosis.

Serum sCD163 levels were quantified by enzyme-linked immunosorbent assay in accordance with the manufacturer's protocol (eBioscience, USA). The samples were obtained at the time of PM/DM-related ILD diagnosis and stored at −80 °C for future analysis. All measurements were done in duplicate and the mean value of serum sCD163 was recorded. Written informed consent for serum conservation was obtained from all the subjects.

### Immunohistochemical analysis

Lung specimens were obtained from two patients with classic DM-related ILD (on surgical lung biopsy and autopsy, respectively) and one patient with early-stage lung cancer as a normal control (on lung resection). These specimens had been fixed in 10% formalin and embedded in paraffin. Deparaffinized sections (5 μm thick) were preheated to 120 °C for 20 minutes. After blocking endogenous peroxidase activity with 0.3% H_2_O_2_ for 20 minutes, the slides were incubated with the rabbit anti-human CD163 monoclonal antibody (1:200; Leica Biosystems, Germany) or isotype control antibody (Jackson ImmunoResearch Laboratories, USA) for 1 h at 20 °C. Subsequently, the sections were incubated with biotin-conjugated gout anti-rabbit IgG antibody (Nichirei, Japan) for 15 minutes, and the immunoreaction was visualized using a 3,3-diaminobenzidine (DAB) chromogen solution (DAB substrate kit; Vector Laboratories, USA), and then counterstained with hematoxylin. Additionally, hematoxylin–eosin staining was also done on another slide from each patient.

### Statistical analysis

Data were described as numbers (percentages) or medians with observed ranges. Spearman's rank correlation coefficients were evaluated for correlation between serum sCD163 and the other clinical factors with continuous variables. The Mann–Whitney *U* test was performed for two-group comparisons. One-way analysis of variance was performed followed by post-hoc analysis (Dunn's multiple comparisons test) for multi-group comparisons. Overall survival time was defined as the number of months from PM/DM-related ILD diagnosis (the date of the first HRCT evaluation) until all-cause death or at censoring. Patients were censored if alive on 30 June 2015. Cox proportional hazard modeling was used to identify prognostic factors. To estimate the cutoff value of serum sCD163 level, time-dependent receiver operating characteristic analysis was performed, as described previously [25, 26]. The Kaplan–Meier survival method and the log-rank test were employed to compare the survival rate between groups. Values of *P* < 0.05 were considered significant. Statistical analyses were performed by using R software version 2.15.1 (The R Foundation for Statistical Computing, Austria) and SPSS software version 13.0 (SPSS, USA).

## Results

### Baseline characteristics

Baseline characteristics of patients with PM/DM-related ILD are summarized in Table 1. The numbers of patients diagnosed with classic DM, CADM, and PM were 24, 21, and 3, respectively. Twenty patients (42%) had a smoking history. The median serum levels of CK and C-reactive protein (CRP) were almost within normal limits. Serum ferritin levels were highly variable, ranging from 14 ng/mL to 12,701 ng/mL. Using immunoprecipitation methods, anti-melanoma differentiation-associated genes 5 (MDA5) antibody and anti-aminoacyl-tRNA synthetase (ARS) antibody were detected in 14 patients (29%) and 19 patients (40%), respectively. Mildly decreased values of arterial partial pressure of oxygen (PaO_2_), percent predicted FVC, and percent predicted DLco were commonly observed. The BAL fluid revealed a slightly high percentage of lymphocytes (median 7.2%, range 1.2–70.0%). All patients received radiological examinations using chest HRCT, in which bilateral consolidation and/or ground glass attenuation were commonly shown.

**Table 1:** Baseline characteristics and the correlations with serum soluble CD163 levels in patients with polymyositis/dermatomyositis-related interstitial lung disease

Variables	Patients (n = 48)	*r*	*P* value
Age at the ILD diagnosis (years)	55, 32–76	−0.05	0.72
Male	17 (35%)	−	−
Current or former smoker	20 (42%)	−	−
CADM/classic DM/PM	21 (44%)/24 (50%)/3 (6%)	−	−
Fever	20 (42%)	−	−
Cough	20 (42%)	−	−
Dyspnea	23 (48%)	−	−
Muscle pain or weakness	27 (56%)	−	−
Rash typical of DM	45 (94%)	−	−
Raynaud’s phenomenon	5 (10%)	−	−
Arthralgia	17 (35%)	−	−
CK (IU/mL)	162, 24–5274	0.24	0.10
CRP (mg/dL)	0.36, 0.03–8.33	0.32	0.03
KL-6 (U/mL)	883, 249–4323	0.14	0.33
Ferritin (ng/mL)	116.5, 14–12701	0.25	0.08
PaO_2_ on room air (Torr)	75.3, 47.9–109.0	−0.16	0.28
Anti-MDA5-positive/anti-ARS positive/others	14 (29%)/19 (40%)/15 (31%)	−	−
Predicted FVC (%)	65.8, 40.6–107.7 (n = 45)	−0.30	0.045
Predicted DLco (%)	60.3, 27.4–127.2 (n = 18)	−0.15	0.55
BAL-lymphocytes (%)	7.2, 1.2–70.0 (n = 35)	0.03	0.85

Data are presented as number (%) or median, range. All *P* values are derived from the Spearman's rank correlation coefficient. *ARS* aminoacyl-tRNA synthetase, *BAL* bronchoalveolar lavage, *CADM* clinically amyopathic dermatomyositis, *CK* creatine kinase, *CRP* C-reactive protein, *DLco* diffusing capacity of the lung for carbon monoxide, *DM* dermatomyositis, *FVC* forced vital capacity, *ILD* interstitial lung disease, *KL-6* Krebs von den Lungen-6, *PaO2* arterial partial pressure of oxygen, *MDA5* melanoma differentiation-associated gene 5, *PM*, polymyositis

### Serum sCD163

**Fig. 1:**
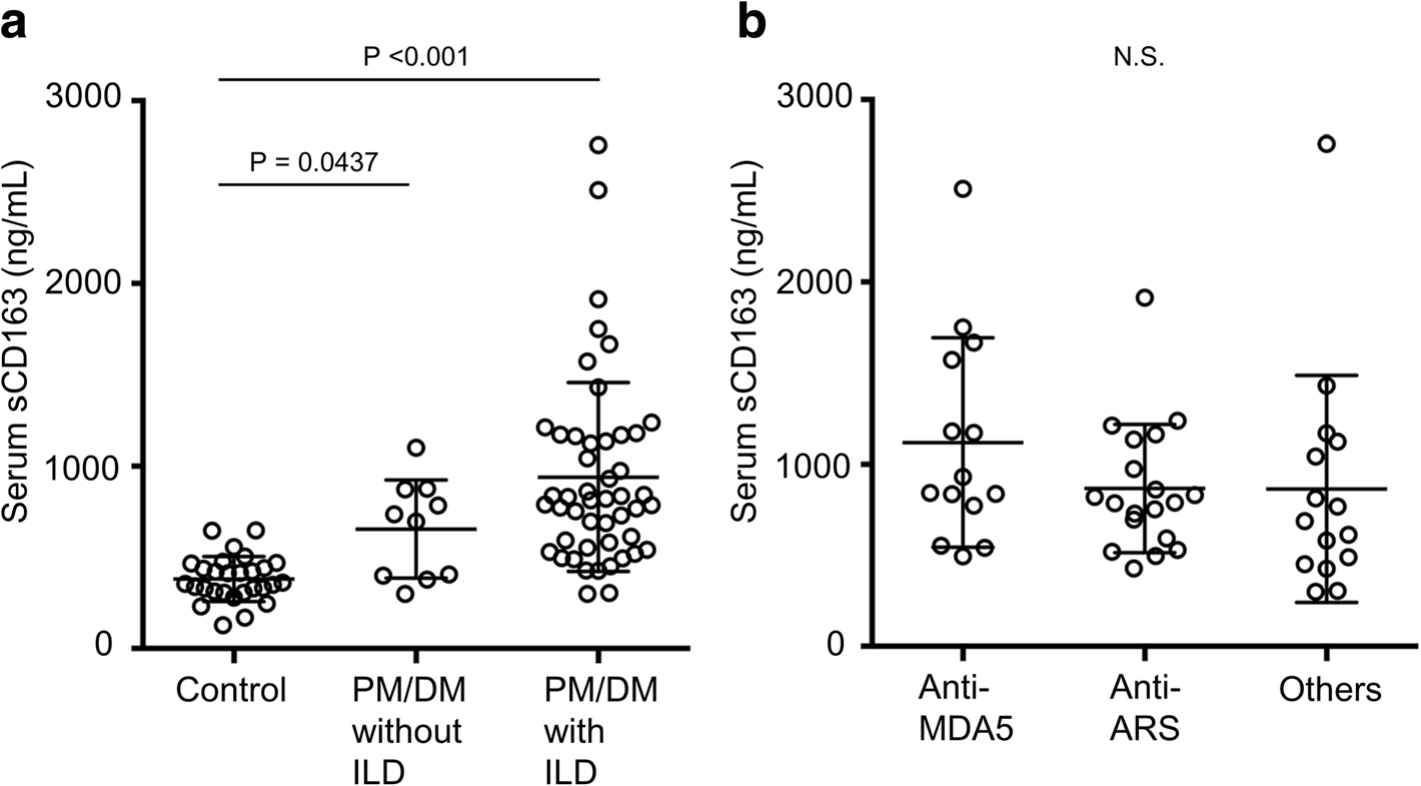
Comparison of serum soluble CD163 levels. **a** Patients with polymyositis (*PM*)/dermatomyositis (*DM*)-related interstitial lung disease (*ILD*) (n = 48) vs patients with PM/DM without ILD (n = 10) vs healthy volunteers (n = 20). **b** Patients with PM/DM-related ILD with anti-melanoma differentiation-associated gene 5 (*MDA5*) antibody (n = 14) vs those with anti-aminoacyl-tRNA synthetase (*ARS*) antibody (n = 19) vs those without both antibodies (n = 15). One-way analysis of variance followed by post-hoc analysis (Dunn's multiple comparisons test) was used for multi-group comparisons

Serum concentrations of sCD163 are compared in Fig. 1a. The median value of serum sCD163 was 818 ng/mL (range 300–2759 ng/mL) in patients with PM/DM-related ILD, which was relatively higher than that in patients with PM/DM without ILD (median 716 ng/mL, range 250–1101 ng/mL). Patients with PM/DM, regardless of the presence/absence of ILD, had significantly higher sCD163 levels than healthy volunteers (median 340 ng/mL, range 127–647 ng/mL).

Table 1 shows the correlation between serum sCD163 levels and other clinical factors at PM/DM-related ILD diagnosis. Although the coefficients were generally low, serum sCD163 was positively correlated with serum CRP (*r* = 0.322, *P* = 0.03) and negatively correlated with % predicted FVC (*r* = −0.301, *P* = 0.045). There was weak, non-significant correlation with serum ferritin (*r* = 0.25, *P* = 0.08). Patients with fever and arthralgia had significantly higher sCD163 than those without such symptoms (Table 2). The comparison of sCD163 levels according to the profile of myositis-specific autoantibodies is shown in Fig. 1b. Although not significant, patients with anti-MDA5 antibody exhibited relatively higher values.

**Table 2:** Comparison between baseline categorical variables and serum soluble CD163 levels in patients with polymyositis/dermatomyositis-related interstitial lung disease

Variables	Soluble CD163, ng/mL	*P* value
Male/female	814 (300–1572)/822 (305–2759)	0.66
Current or former smoker/never smoker	791 (300–2758)/834 (305–2512)	0.39
CADM/others	730 (305–1750)/862 (300–2759)	0.09
Fever, yes/no	1009 (428–2512)/742 (300–2759)	0.03
Cough, yes/no	806 (426–1572)/826 (300–2759)	0.59
Dyspnea, yes/no	814 (428–2759)/822 (300–1572)	0.48
Muscle pain or weakness, yes/no	862 (300–2759)/730 (305–1750)	0.09
Rash typical of DM, yes/no	789 (300–2759)/1124 (814–1213)	0.30
Raynaud’s phenomenon, yes/no	593 (426–1137)/822 (300–2759)	0.27
Arthralgia, yes/no	1124 (305–2512)/773 (300–2759)	0.02
Anti-MDA5 antibody, positive/negative	887 (495–2512)/778 (300–2759)	0.09

Data are presented as median (observed range). All *P* values are derived from the Mann–Whitney *U* test. *CADM* clinically amyopathic dermatomyositis, *DM* dermatomyositis, *MDA5* Melanoma differentiation-associated gene 5

### Clinical course

The median follow-up period in patients with PM/DM-related ILD was 85.2 months (range 5.8–211.4 months). Only two patients were lost to follow up. Most patients (46/48, 96%) received immunosuppressive therapy after ILD diagnosis. Among these patients, combination regimens of high-dose corticosteroids and immunosuppresssants, including cyclophosphamide, cyclosporine, and tacrolimus, were administered to 38 patients; in contrast, corticosteroid monotherapy was selected for the other 8 patients. Although most patients had a favorable response to the treatment, 13 patients died during the follow-up period. The most common cause of death was respiratory failure due to ILD (n = 9), followed by cancer of unknown primary origin (n = 1), myocarditis (n = 1), rupture of aortic aneurysm (n = 1), and unknown cause (n = 1). Among the non-surviving patients, lung specimens were available in three patients, which showed diffuse alveolar damage on surgical lung biopsy (n = 1) or autopsy (n = 1) and usual interstitial pneumonia with moderate lymphocytic alveolitis on surgical lung biopsy (n = 1).

**Fig. 2:**
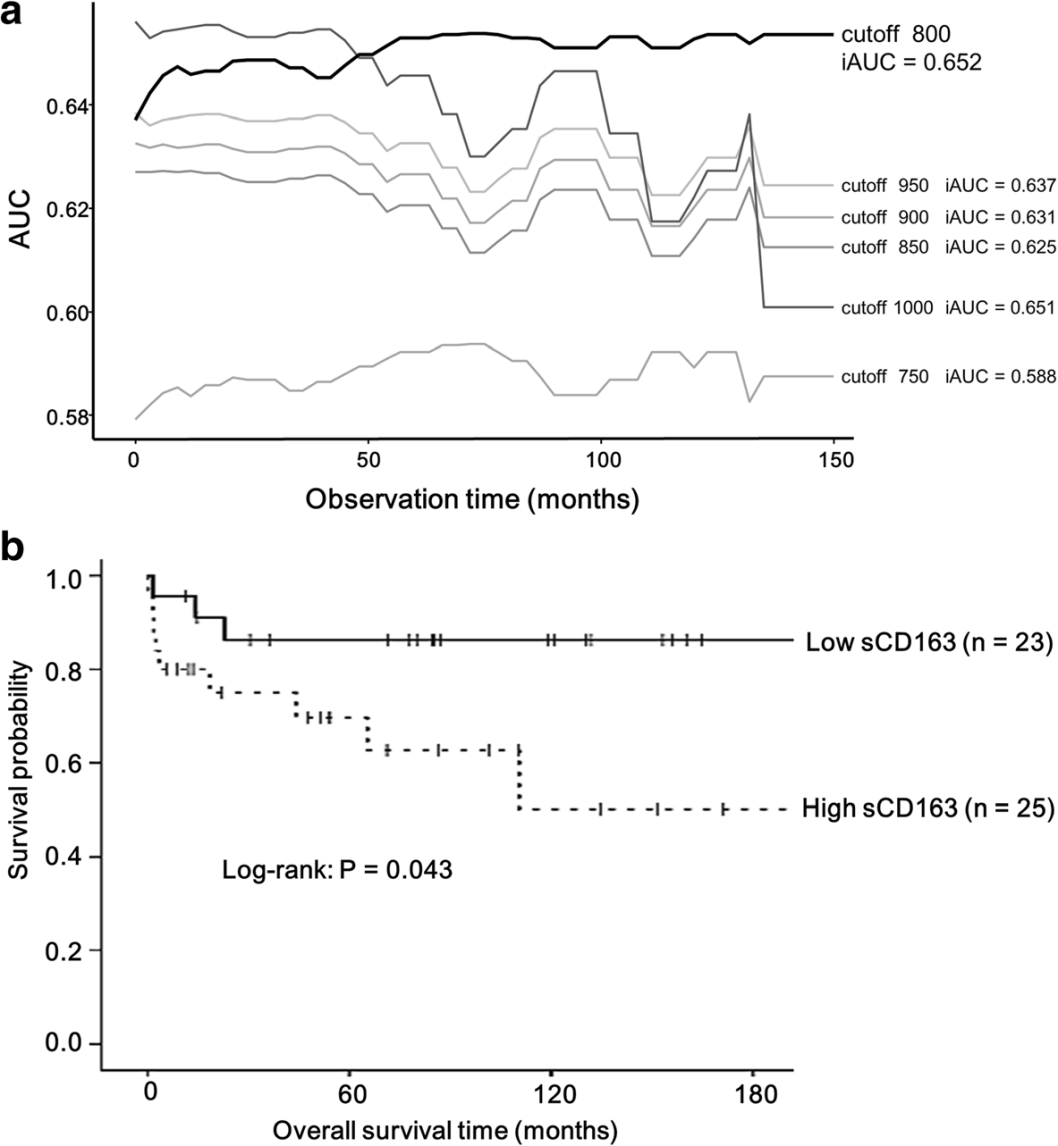
Prognostic analysis. **a** Estimation of the cutoff for serum soluble CD163. **b** Survival curves in patients with polymyositis/dermatomyositis-related interstitial lung disease. The cutoff for serum soluble CD163 is set at 800 ng/mL by comparing the integrated area under the curve (*iAUC*)

The results of the univariate Cox proportional hazard model are listed in Table 3. Among the baseline clinical factors, presence of fever, higher level of serum ferritin, higher level of serum sCD163, lower PaO_2_, and lower percent predicted FVC were significantly associated with worse prognosis. The age-adjusted and gender-adjusted hazard ratio for serum sCD163 (per 100 ng/mL increase) was 1.27 (95% confidence interval 1.11–1.45, *P* < 0.001). Additionally, even when classifying the four patients whose cause of death was not respiratory failure due to ILD into censored cases, the prognostic significance of serum sCD163 remained (age-adjusted and gender-adjusted hazard ratio 1.26, 95% confidence interval 1.09–1.45, *P* = 0.002). As shown in Fig. 2a, the cutoff for survival was calculated as 800 ng/mL. The comparisons of baseline characteristics between patients with higher and lower values of serum sCD163 are summarized in Additional file 1: Table S2. The patients with higher serum sCD163 had significantly lower survival rates than those with lower serum sCD163 (log rank test, *P* = 0.043) (Fig. 2b).

**Table 3:** Analysis of prognostic factors in patients with polymyositis/dermatomyositis-related interstitial lung disease using the Cox proportional hazard model

Variables	Unadjusted HR	95% CI	*P*	Age-adjusted and gender-adjusted HR	95% CI	*P*
Age (per 1 year increase)	1.01	0.96–1.07	0.62	−	−	−
Male	1.93	0.61–6.09	0.26	−	−	−
Current or former smoker	2.27	0.71–7.23	0.17	1.96	0.47–8.24	0.36
CADM	0.37	0.10–1.38	0.14	0.41	0.11–1.60	0.12
Fever	4.29	1.27–14.50	0.02	4.68	1.36–16.10	0.01
Cough	0.65	0.20–2.16	0.48	0.76	0.22–2.67	0.67
Dyspnea	2.30	0.69–7.64	0.18	2.65	0.78–9.03	0.12
Muscle pain or weakness	2.69	0.73–9.97	0.14	2.44	0.63–9.48	0.20
Rash typical for DM	0.40	0.09–1.85	0.24	0.47	0.10–2.27	0.35
Raynaud’s phenomenon	2.99	0.81–11.13	0.10	3.03	0.72–12.84	0.13
Arthralgia	1.54	0.49–4.90	0.46	1.39	0.42–4.59	0.59
CK (per 10 IU/mL increase)	1.00	0.995–1.01	0.97	1.00	0.994–1.006	0.96
CRP (per 1 mg/dL increase)	1.13	0.91–1.41	0.27	1.13	0.91–1.41	0.27
KL-6 (per 100 U/mL increase)	1.06	0.998–1.12	0.06	1.05	0.995–1.12	0.07
Ferritin (per 100 ng/mL increase)	1.02	1.01–1.04	0.01	1.03	1.01–1.05	0.01
Soluble CD163 (per 100 ng/mL increase)	1.22	1.09–1.36	0.001	1.27	1.11–1.45	<0.001
PaO_2_ on room air (per 10 Torr increase)	0.48	0.30–0.76	0.002	0.48	0.28–0.81	0.01
Anti-MDA5-positive	2.60	0.81–8.32	0.11	3.11	0.90–10.74	0.07
Percent predicted FVC (per 10% increase)	0.73	0.61–0.88	0.001	0.65	0.50–0.83	0.001

*ARS* aminoacyl-tRNA synthetase, *CADM* clinically amyopathic dermatomyositis, *CI* confidence interval, *CK* creatine kinase, *CRP* C-reactive protein, *DM* dermatomyositis, *FVC* forced vital capacity, *HR* hazard ratio, *KL-6* Krebs von den Lungen-6, *MDA5* melanoma differentiation-associated gene 5

### Immunohistochemical findings

**Fig. 3:**
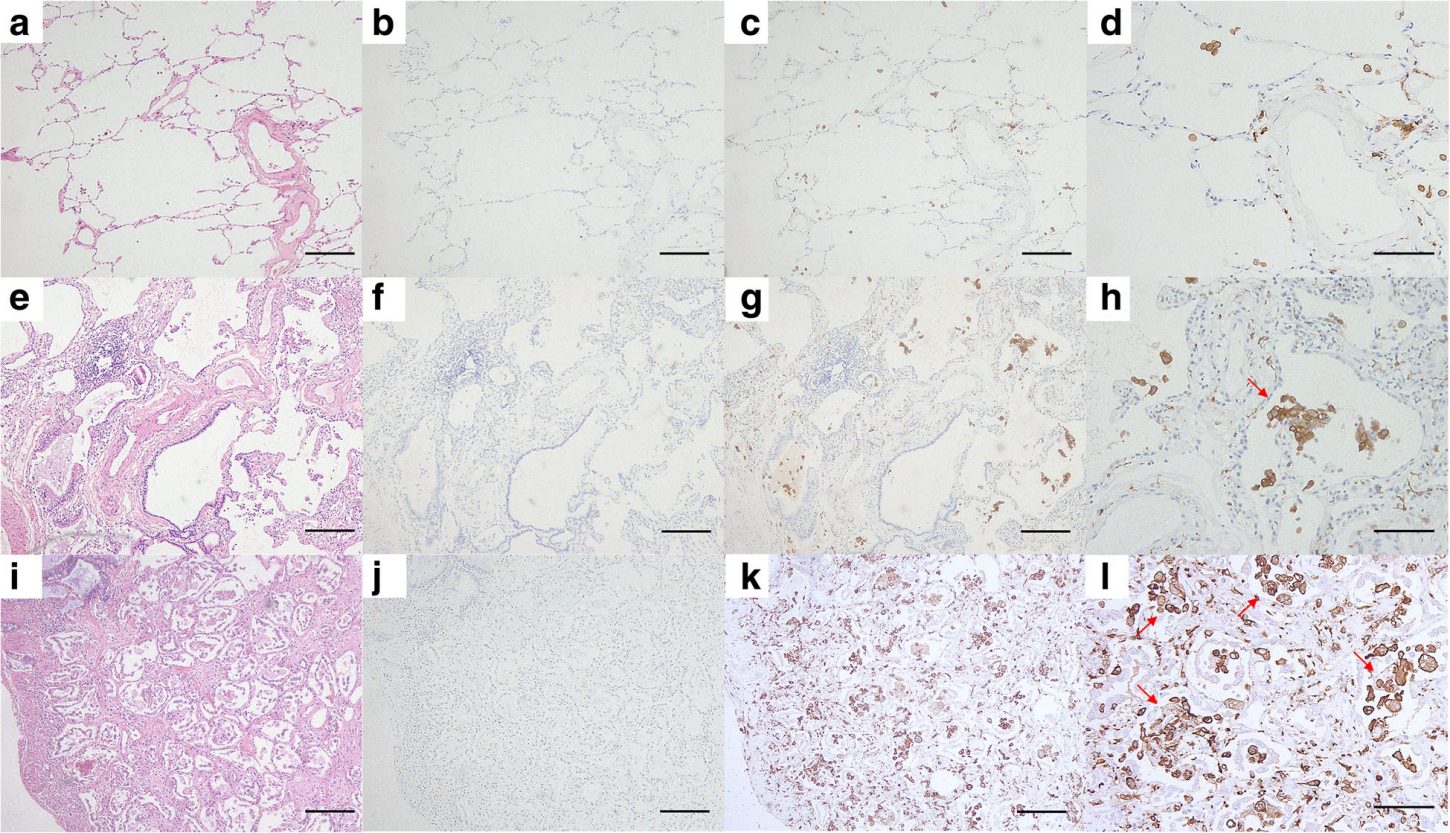
Immunohistochemical staining. Lung sections from an early lung cancer patient (**a**–**d**), a surviving patient with classic dermatomyositis (DM)-related interstitial lung disease (ILD) (**e**–**h**: 66 year-old male patient, serum sCD163 = 519 ng/mL), and a non-surviving patient with classic DM-related ILD (**i**–**l**: 32 year-old male patient, serum sCD163 = 1572 ng/mL) are shown. CD163-positive macrophages are evident in the lungs of the two patients with classic DM-related ILD, especially in lung of the non-survivor. Accumulations of CD163-positive alveolar macrophages are observed (*arrows*). **a**, **e**, and **i** Hematoxylin − eosin staining; scale bar 200 μm. **b**, **f**, and **j** Isotype controls, scale bar 200 μm. **c**, **g**, and **k** Staining with anti-CD163 antibody with lower magnification (×40), scale bar 200 μm. **d**, **h**, and **l** Staining with anti-CD163 antibody with higher magnification (×100), scale bar 100 μm

The immunohistochemical results for lung sections are shown in Fig. 3. CD163 expression in resected lung sections from an early lung cancer patient (Fig. 3a–d), a surviving patient with classic DM-related ILD (Fig. 3e–h; 66 year-old male patient, serum sCD163 = 519 ng/mL), and a non-surviving patient with classic DM-related ILD (Fig. 3i–l; 32 year-old male patient, serum sCD163 = 1572 ng/mL) were evaluated. Compared with normal lung, accumulation of CD163-positive macrophages at alveolar spaces was more evident in the lungs of the two patients with classic DM-related ILD. Especially, the macrophage infiltration in the non-survivor’s lung was apparently severe, mainly at alveolar spaces and partly at alveolar walls.

## Discussion

In this study, we evaluated the clinical significance of sCD163 in patients with PM/DM-related ILD. Serum sCD163 levels at ILD diagnosis were associated with several clinical factors and even the prognosis, suggesting that this molecule might be a potential biomarker of the disease.

The expression of CD163, a macrophage-specific marker within the macrophage/monocyte lineage, is intricately regulated by various factors, such as IL-6, IL-10, interferon-γ, tumor necrosis factor (TNF)-α, and corticosteroids. Cleavage of CD163 from activated macrophages, which is performed by a disintegrin and metalloproteinase 17/TNF-α converting enzyme in response to several inflammatory mediators, leads to an increase in sCD163 in the serum [10, 11]. In fact, the serum sCD163 levels in our patients were correlated with the presence of fever and serum CRP levels, suggesting that elevated serum sCD163 is associated with systemic inflammation. Notably, among macrophages, CD163 is mainly expressed on alternatively activated macrophages, rather than classically activated macrophages [11]. Collectively, our results indicate the significance of alternatively activated macrophages in the inflammation seen in PM/DM-related ILD.

Serum sCD163 levels were significantly higher in patients with PM/DM-related ILD than those in healthy volunteers, which is consistent with the results of the recent study by Peng et al. [13]. They measured serum sCD163 levels in 108 patients with PM/DM, consisted of 65 patients without ILD and 43 patients with ILD, and found that serum sCD163 levels were correlated with the activity of myositis. However, the clinical implication of sCD163 in PM/DM-related ILD remains unclear. In the present study, we focused on the ILD and further advanced the understandings of sCD163 physiology in this disease entity, particularly the relevance to pulmonary function and prognosis.

In the Cox proportional hazard modeling, higher serum sCD163 was significantly associated with worse prognosis. This result implies that macrophage activation in PM/DM-related ILD plays an important role in advancing the disease. In contrast, several studies have evaluated the clinical significance of serum ferritin, which is a key biomarker of MAS, in PM/DM-related ILD [27–29]. Indeed, PM/DM is known to cause MAS, in which the lack of T cell regulation and excessive production of cytokines, including TNF-α, IL-1β, IL-6, and IL-18, results in macrophage activation. Those previous studies reported that hyperferritinemia was associated with high mortality in patients with PM/DM-related (particularly CADM-related) ILD, which was reproduced in our cohort. Interestingly, there was a weak, but not significant correlation between serum sCD163 levels and ferritin levels in the present study. The total value of serum ferritin might be affected by the storage of iron (in the form of L-subunit ferritin). Meanwhile, sCD163 is secreted from activated macrophages and elevated for days in peripheral blood, suggesting that this molecule specifically reflects the degree of macrophage activation. Although the superiority of either of the two biomarkers could not be evaluated in our small cohort, our results suggest that serum sCD163 may have value in the assessment of disease severity in PM/DM-related ILD.

Human CD163 expression has been confirmed in several tissues, such as red pulp macrophages, bone marrow macrophages, liver macrophages (Kupffer cells), and alveolar macrophages [10, 11]. In fact, on the basis of immunohistochemical analysis, one of the sources of elevated serum sCD163 seemed to be the diseased lungs. Additionally, more severe infiltration of CD163-positive macrophages was found in the lung of the non-surviving patient with DM-related ILD, compared with that in the lung of the survivor (Fig. 3). Although we are speculating on the assessment of only two lung specimens, the amount of infiltrating CD163-positive macrophage may reflect the severity of PM/DM-related ILD. The following results may support this: serum sCD163 was negatively correlated with percent predicted FVC. Previous studies demonstrate that alveolar macrophages produce several profibrotic cytokines including transforming growth factor-β, platelet-derived growth factor, and fibroblast growth factor-2 [16, 30, 31]. Intriguingly, Gibbons et al. recently demonstrated the paradoxical and essential role played by macrophages in the pathogenesis of lung fibrosis, in which activated macrophages had both profibrotic and resolution-promoting roles during fibrogenesis and the reversible phase, respectively [17]. Thus, the exact functions of CD163-positive macrophages in the pathogenesis of PM/DM-related ILD remain unclear, but the infiltration to the lungs may have some impact not only on inflammation but also on fibrosis.

The present study has several limitations. First, this was a small, retrospective study, which may have involved various biases. Validation in larger and prospective cohorts is needed to confirm our preliminary results. Second, there are several subtypes in the development of PM/DM-related ILD, such as ILD-preceding, concomitant, and myositis-preceding patterns, which indicates the difficulty in determining the exact ILD onset, particularly in patients with no symptoms or only minor symptoms. Our definition of ILD onset might be not accurate in some patients. Third, the patients included in the present study were not treated according to a consistent regimen. Although most of the patients were treated with corticosteroids and additional immunosuppressants, regimen variations, such as the doses and selection of drugs, may have affected the clinical disease course and prognosis. Additionally, we could not follow serum sCD163 levels chronologically. It is unclear how changes correlated with the clinical course of PM/DM-related ILD. In future studies, clinical factors important in the management of ILD, such as changes in pulmonary function and oxygenation, and treatment response, should be compared with changes in sCD163 levels. These would explore the usefulness of this biomarker.

## Conclusions

Serum sCD163 might have potential for predicting the disease severity and prognosis in PM/DM-related ILD. Our results suggest the involvement of macrophage activation in this disease.

## Additional file

Additional file 1: Table S1. Baseline characteristics of all subjects included in this study. **Table S2.** Comparison of baseline characteristics between patients with higher and lower values of serum soluble CD163 (cutoff 800 ng/mL). (DOCX 45 kb)

## Abbreviations

ARS: aminoacyl-tRNA synthetase
BAL: bronchoalveolar lavage
CADM: clinically amyopathic dermatomyositis
CK: creatine kinase
CRP: C-reactive protein
DLco: diffusing capacity of the lung for carbon monoxide
DM: dermatomyositis
FVC: forced vital capacity
HRCT: high-resolution computed tomography
IL: interleukin
ILD: interstitial lung disease
MAS: macrophage activation syndrome
MDA5: Melanoma differentiation-associated gene 5
PaO2: arterial partial pressure of oxygen
PM: polymyositis
sCD163: soluble CD163
TNF: tumor necrosis factor
