# Supplementary material for: Clinical significance of soluble CD163 in polymyositis-related or dermatomyositis-related interstitial lung disease
Source: Arthritis Res Ther. 2017 Jan 19;19:9. doi: 10.1186/s13075-016-1214-8 (PMC5248519; doi:10.1186/s13075-016-1214-8)
Supplement: Additional file 1: Table S1. — Baseline characteristics of all subjects included in this study. Table S2. Comparison of baseline characteristics between patients with higher and lower values of serum soluble CD163 (cutoff 800 ng/mL). (DOCX 45 kb) [file 13075_2016_1214_MOESM1_ESM.docx]

**Table S1**. Baseline characteristics of all subjects included in this study

|  | PM/DM with ILD (n = 48) | PM/DM without ILD (n = 10) | Healthy controls (n = 20) |
| --- | --- | --- | --- |
| Age | 55, 32–76 | 54.5, 44–83 | 56.5, 26–63 |
| Male | 17 (35%) | 3 (30%) | 7 (35%) |
| Current or former smoker | 20 (42%) | 4 (40%) | 9 (45%) |

Data are presented as number (%) or median with observed range.

DM, dermatomyositis; ILD, interstitial lung disease; PM, polymyositis.

**Table S2.** Comparison of baseline characteristics between patients with higher and lower values of serum soluble CD163 (cut off: 800 ng/mL)

| Variables | High (n = 25) | Low (n = 23) | *P*-value |
| --- | --- | --- | --- |
| Age at the ILD diagnosis (years) | 56, 32–68 | 54, 38–76 | 0.85 |
| Male | 9 (36%) | 8 (35%) | 1.00 |
| Current or former smoker | 10 (40%) | 10 (43%) | 1.00 |
| CADM/classic DM/PM | 8 (32%)/14 (56%)/3 (12%) | 13 (57%)/10 (43%)/0 (0%) | 0.09 |
| Fever | 14 (56%) | 6 (26%) | 0.045 |
| Cough | 10 (40%) | 10 (43%) | 1.00 |
| Dyspnea | 12 (48%) | 11 (48%) | 1.00 |
| Muscle pain or weakness | 17 (68%) | 10 (43%) | 0.15 |
| Rash typical for DM | 22 (88%) | 23 (100%) | 0.24 |
| Raynaud’s phenomenon | 2 (8%) | 3 (13%) | 0.66 |
| Arthralgia | 12 (48%) | 5 (22%) | 0.08 |
| CK (IU/mL) | 222, 33–5274 | 89, 24–4767 | 0.07 |
| CRP (mg/dL) | 0.42, 0.03–8.33 | 0.20, 0.04–4.78 | 0.29 |
| KL-6 (U/mL) | 888, 249–4323 | 878, 254–2660 | 0.93 |
| Ferritin (ng/mL) | 218, 16–12701 | 87, 14–1480 | 0.15 |
| PaO_2_ on room air (Torr) | 76.0, 47.9–109.0 | 74.5, 61.0–105.0 | 0.42 |
| Anti-MDA5-positive/Anti-ARS positive/Others | 10 (40%)/9 (36%)/6 (24%) | 4 (17%)/10 (43%)/9 (39%) | 0.21 |
| % predicted FVC (%) | 64.7, 40.9–98.9 (n = 24) | 66.2, 40.6–107.7 (n = 21) | 0.29 |
| % predicted DLco (%) | 54.6, 36.3–127.2 (n = 11) | 62.3, 27.4–81.4 (n = 7) | 0.96 |
| BAL-lymphocytes (%) | 9.0, 1.6–70.0 (n = 20) | 6.2, 1.2–60.0 (n =15) | 0.44 |

Data are presented as number (%) or median with observed range. All *P*-values are evaluated using Fisher’s exact test, chi-squared test, and Mann–Whitney’s *U* test as appropriate.

ARS, aminoacyl-tRNA synthetase; BAL, bronchoalveolar lavage; CADM, clinically amyopathic dermatomyositis; CK, creatine kinase; CRP, C-reactive protein; DLco, diffusing capacity of the lung for carbon monoxide; DM, dermatomyositis; FVC, forced vital capacity; ILD, interstitial lung disease; KL-6, Krebs von den Lungen-6; MDA5, melanoma differentiation-associated gene 5; PM, polymyositis.
